# Drug-Induced Hyperprolactinemia and Granulomatous Mastitis: A Case Report and Literature Review

**DOI:** 10.1155/crdi/9409072

**Published:** 2025-05-29

**Authors:** Mohammed Alenazi, Sarah Howse, Syed Ali Imran

**Affiliations:** ^1^Division of Endocrinology and Metabolism, Dalhousie University, Halifax, Nova Scotia, Canada; ^2^Diabetes and Endocrine Treatment Center, Prince Sultan Military Medical City, Riyadh, Saudi Arabia

## Abstract

Granulomatous mastitis (GM) is a rare inflammatory condition of the breast that can mimic inflammatory breast cancer. We report a case of a 54-year-old female who developed recurrent GM symptoms in the context of drug-induced hyperprolactinemia, which resolved with dopamine agonist therapy. Our report suggests that serum prolactin should be tested in patients with GM and appropriately managed if elevated.

## 1. Introduction

Granulomatous mastitis (GM) is a rare inflammatory condition of the breast [1]. It was first described in 1972 as a chronic inflammatory disorder that typically affects women of childbearing age, particularly those with a recent history of pregnancy or breastfeeding [2]. The clinical presentation of GM often mimics inflammatory breast cancer and can only be reliably distinguished through histopathology [1].

While the association between hyperprolactinemia and GM is not fully understood, elevated prolactin (PRL) levels have been linked to recurrent GM and a prolonged disease course [3]. There is no universally accepted treatment for GM; however, therapeutic options include antibiotics, wide surgical excision, immunosuppressants, and corticosteroids [4]. In cases of recurrent GM, dopamine agonists (DAs) have been attempted due to the growing recognition of the correlation between GM and hyperprolactinemia [5].

Several reported cases have linked GM to hyperprolactinemia, either induced by medications such as antipsychotics or secondary to a prolactinoma. This case highlights the importance of considering hyperprolactinemia as a potential predisposing factor for GM and aims to raise awareness among healthcare providers regarding the risk of developing GM in patients with chronic, uncontrolled hyperprolactinemia.

## 2. Case Presentation

**Figure 1:**
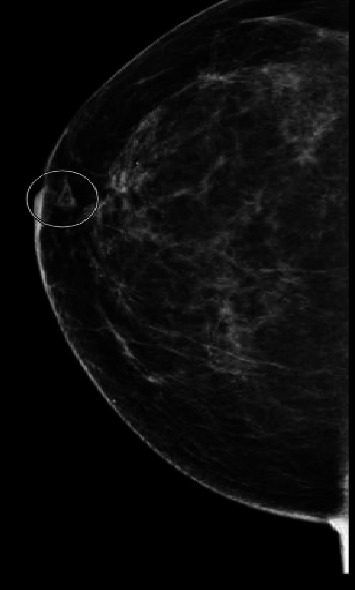
Focal asymmetry anteriorly at 11 O'clock position of the right breast.

**Figure 2:**
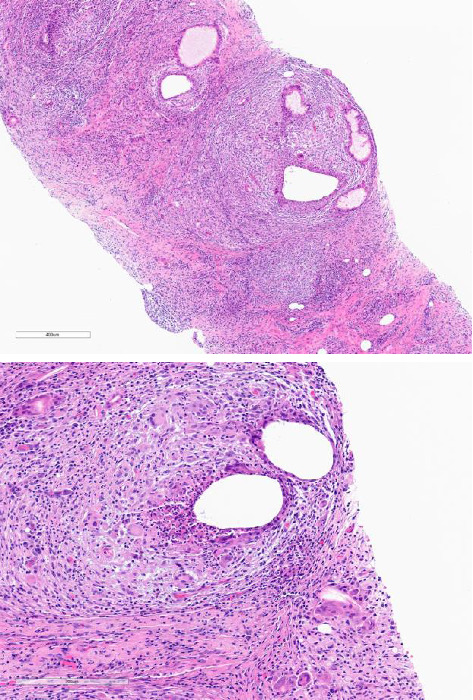
Microscopic findings of granulomatous mastitis. There are empty spaces of varying sizes surrounded by granulomatous inflammation.

A 54-year-old female with a history of fibromyalgia, hypothyroidism, depression, anxiety, and chronic hyperprolactinemia presented to the infectious disease clinic in 2019 with a 4-month history of a warm, erythematous lump in her right breast. Her family physician prescribed a course of cephalexin and referred her for further evaluation. A mammogram revealed a suspicious lesion (Figure 1), prompting a biopsy that showed “acute and chronic granulomatous inflammation” with “Gram-positive cocci” on the stain (Figure 2). She responded to antibiotics, and her swelling and pain subsided.

**Figure 3:**
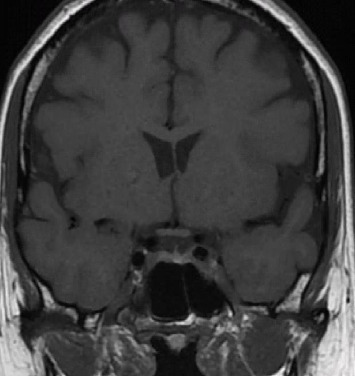
MRI sella—T1 coronal view showed small pituitary microadenoma.

In 2020, she was referred to the pituitary clinic due to persistently elevated PRL levels. She had experienced hyperprolactinemia for at least 12 years, along with occasional whitish breast discharge from the right breast, which had previously been attributed to opioid therapy for pain. Her initial serum PRL level was 81 μg/L (normal range: 5–25 μg/L). An MRI of the sella showed mild stalk deviation to the left with a small hypoenhancing lesion measuring 5 × 7 mm in the right lateral sella (Figure 3). She declined DA therapy due to concerns about potential side effects and was instead monitored periodically. She also declined to discontinue opioid therapy.

Six months later, she experienced a recurrence of breast discomfort and returned to the infectious disease clinic with symptoms suggestive of mastitis. She was prescribed a 2-month course of doxycycline, but her symptoms failed to resolve. Due to persistently elevated PRL levels (ranging between 63 and 81 μg/L) and unresolved mastitis, she was prescribed cabergoline 0.5 mg once a week. Her symptoms rapidly improved with therapy, and she has remained asymptomatic since, with no further recurrence of mastitis. Follow-up MRI findings remained unchanged, and at her most recent visit, her PRL level was 6.3 μg/L. She continues to tolerate DA therapy well.

## 3. Discussion

GM is a rare, benign inflammatory condition of the breast [2]. The first case series of GM was reported by Kessler and Wolloch in 1972, describing five patients who underwent surgery for suspected malignant tumors but were later found to have multiple noncaseating granulomas and abscess formation on histopathology [2]. Since then, numerous cases of GM have been reported [6–10].

Over time, the disease, initially termed *idiopathic GM*, has been categorized into primary (idiopathic) and secondary (infectious and noninfectious) GM [11]. Although the exact pathogenesis and etiology remain unclear, potential causes include autoimmune responses, infectious agents, and hormonal disorders [12].

One potential predisposing factor of GM is hyperprolactinemia. It is believed that milk stasis plays a crucial role in GM, explaining the correlation between GM, breastfeeding, and pregnancy [13]. Furthermore, PRL may trigger an inflammatory response in the breast [3, 14]. A retrospective study found that elevated serum PRL levels were an independent risk factor for GM recurrence, with persistently elevated PRL increasing the likelihood of recurrence [15].

DAs have been used both to treat hyperprolactinemia and, in some cases, as a therapeutic trial to reduce GM recurrence [5, 16]. This approach has minimized the need for surgery and reduced recurrence rates. Previously, only three cases of drug-induced hyperprolactinemia and GM have been reported [3, 17, 18], whereas other cases involved underlying prolactinomas [19–21]. Given the large number of patients using medications that increase PRL, there may be more unrecognized cases. However, many GM studies do not report PRL levels, making it difficult to determine the true extent of such cases. A summary of relevant literature on GM and hyperprolactinemia is presented in Table 1.

## 4. Conclusion

This case report underscores the importance of recognizing hyperprolactinemia as a potential predisposing factor in the development of GM. Endocrinologists should educate patients about this disorder in cases of longstanding hyperprolactinemia and maintain a low threshold for treating hyperprolactinemia in patients presenting with GM [26].

## Figures and Tables

**Table 1:** Literature review summary.

**Case reports**					
	**Case**	**Age**	**Prolactin level**	**Recurrence**	**Follow-up**

Destek et al., 2017, Turkey [21]	PRLoma	39	351 ng/m	No	4 years
Lin et al., 2012, Taiwan [18]	Drug-induced hyperprolactinemia	39	84.5 ng/mL	No	
Agrawal and Pabolu, 2019, USA [20]	PRLoma	41	405.5 ng/mL	Yes	
Li and McGregor, 2017, Australia [3]	Drug-induced hyperprolactinemia	55	935 mIU/L	No	18 months
Holla et al., 2017, India [17]	Drug-induced hyperprolactinemia	30	59.8 ng/mL	Yes	
Holla et al., 2017, USA [17]	PRLoma	42	200 ng/mL	Yes	6 months

**Case series/studies**					
	**Number of patients**	**Mean age (Y)**	**Key findings**		

Gautierb et al., 2013 [22]	*n* = 11	38.7	1/11 with prolactinoma.		
Neel et al., 2013 [23]	*n* = 23	39.1	1/23 with prolactinoma. 4/23 on antipsychotic medications		
Wong et al., 2017 [24]	*n* = 37	36	12/37 on antipsychotic. 3/37 with prolactinoma. *Corynebacterium kroppenstedtii* found to be predominant in patients on antipsychotic medication		
Tian et al., 2022 [25]	*n* = 19	31	19/19 on antipsychotic medications PRL 35.15–200 ng/mL		

## Data Availability

The data that support the findings of this study are available from the corresponding author upon reasonable request.

## References

[1] Yin Y., Liu X., Meng Q., Han X., Zhang H., Lv Y. (2022). Idiopathic Granulomatous Mastitis: Etiology, Clinical Manifestation, Diagnosis and Treatment. *Journal of Investigative Surgery*.

[2] Kessler E., Wolloch Y. (1972). Granulomatous Mastitis: A Lesion Clinically Simulating Carcinoma. *American Journal of Clinical Pathology*.

[3] Li J., McGregor H. P. (2017). Idiopathic Granulomatous Mastitis Associated with Hyperprolactinemia: A Nonoperative Approach. *Breast Journal*.

[4] Wolfrum A., Kümmel S., Theuerkauf I., Pelz E., Reinisch M. (2018). Granulomatous Mastitis: A Therapeutic and Diagnostic Challenge. *Breast Care*.

[5] Durgun C. (2023). Cabergoline and Low-Dose Steroid Therapy İn Idiopathic Granulomatous Mastitis. *Dicle Tıp Dergisi*.

[6] Metanat S., Soleimani Jobaneh Y., Noori M. (2022). Global Distribution of Idiopathic Granulomatous Mastitis: A Scoping Review. *Arch Breast Cancer*.

[7] Kok K. Y. Y., Telisinghe P. U. (2010). Granulomatous Mastitis: Presentation, Treatment and Outcome in 43 Patients. *The Surgeon*.

[8] Özşen M., Tolunay Ş, Gökgöz M. Ş (2018). Granulomatous Lobular Mastitis: Clinicopathologic Presentation of 90 Cases. *Turk Patoloji Derg*.

[9] Velidedeoglu M., Umman V., Kilic F. (2023). Correction to: Idiopathic Granulomatous Mastitis: Introducing a Diagnostic Algorithm Based on 5 Years of Follow-Up of 152 Cases from Turkey and a Review of the Literature. *Surgery Today*.

[10] Tse G. M. K., Poon C. S. P., Ramachandram K. (2004). Granulomatous Mastitis: a Clinicopathological Review of 26 Cases. *Pathology*.

[11] Yuan Q.-Q., Xiao S.-Y., Farouk O. (2022). Management of Granulomatous Lobular Mastitis: an International Multidisciplinary Consensus (2021 Edition). *Military Medical Research*.

[12] Altintoprak F., Kivilcim T., Ozkan O. V. (2014). Aetiology of Idiopathic Granulomatous Mastitis. *World Journal of Clinical Cases*.

[13] Dilaveri C., Degnim A., Lee C., DeSimone D., Moldoveanu D., Ghosh K. (2024). Idiopathic Granulomatous Mastitis. *Breast Journal*.

[14] Presentation C. (2019). Clinical Case Reports International Hyperprolactinemia Leading to Mammary Duct Ectasia in a Pre-menopausal Female. *Journal of Medicine*.

[15] Huang Y., Wu H. (2021). A Retrospective Analysis of Recurrence Risk Factors for Granulomatous Lobular Mastitis in 130 Patients: More Attention Should Be Paied to Prolactin Level. *Annals of Palliative Medicine*.

[16] Yu H., Wang Q. (2020). Severe Idiopathic Granulomatous Mastitis Treated with Systemic Medication; A Case Report. *Journal of International Medical Research*.

[17] Holla S., Amberkar M. B., Kamath A., Kamalkishore M. K., Ommurugan B. (2017). Risperidone Induced Granulomatous Mastitis Secondary to Hyperprolactinemia in a Non-pregnant Woman-A Rare Case Report in a Bipolar Disorder. *Journal of Clinical and Diagnostic Research*.

[18] Lin C.-H., Hsu C.-W., Tsao T.-Y., Chou J. (2012). Idiopathic Granulomatous Mastitis Associated with Risperidone-Induced Hyperprolactinemia. *Diagnostic Pathology*.

[19] Nikolaev A., Blake C. N., Carlson D. L. (2016). Association between Hyperprolactinemia and Granulomatous Mastitis. *Breast Journal*.

[20] Agrawal A., Pabolu S. (2019). A Rare Case of Idiopathic Granulomatous Mastitis in a Nulliparous Woman with Hyperprolactinemia. *Cureus*.

[21] Destek S., Gul V. O., Ahioglu S., Serin K. R. (2017). Pituitary Adenoma and Hyperprolactinemia Accompanied by Idiopathic Granulomatous Mastitis. *Case Reports in Endocrinology*.

[22] Gautier N., Lalonde L., Tran-Thanh D. (2013). Chronic Granulomatous Mastitis: Imaging, Pathology and Management. *European Journal of Radiology*.

[23] Neel A., Hello M., Cottereau A. (2013). Long-term Outcome in Idiopathic Granulomatous Mastitis: a Western Multicentre Study. *QJM*.

[24] Wong S. C. Y., Poon R. W. S., Chen J. H. K. (2017). Corynebacterium Kroppenstedtii Is an Emerging Cause of Mastitis Especially in Patients with Psychiatric Illness on Antipsychotic Medication. *Open Forum Infectious Diseases*.

[25] Tian C., Wang H., Liu Z., Han X., Ning P. (2022). Characteristics and Management of Granulomatous Lobular Mastitis Associated with Antipsychotics-Induced Hyperprolactinemia. *Breastfeeding Medicine*.

[26] Lai E. C. H., Chan W. C., Ma T. K. F., Tang A. P. Y., Poon C. S. P., Leong H. T. (2005). The Role of Conservative Treatment in Idiopathic Granulomatous Mastitis. *Breast Journal*.

